# Influence of Image TIFF Format and JPEG Compression Level in the Accuracy of the 3D Model and Quality of the Orthophoto in UAV Photogrammetry

**DOI:** 10.3390/jimaging6050030

**Published:** 2020-05-11

**Authors:** Vincenzo Saverio Alfio, Domenica Costantino, Massimiliano Pepe

**Affiliations:** Dipartimento di Ingegneria Civile, Ambientale, del Territorio, Edile e di Chimica, Polytechnic of Bari, via E. Orabona 4, 70125 Bari, Italy; vincenzosaverio.alfio@poliba.it (V.S.A.); massimiliano.pepe@poliba.it (M.P.)

**Keywords:** UAV, compression, point cloud, photogrammetry, orthophoto, SfM software

## Abstract

The aim of this study is to evaluate the degradation of the accuracy and quality of the images in relation to the TIFF format and the different compression level of the JPEG format compared to the raw images acquired by UAV platform. Experiments were carried out using DJI Mavic 2 Pro and Hasselblad L1D-20c camera on three test sites. Post-processing of images was performed using software based on structure from motion and multi-view stereo approaches. The results show a slight influence of image format and compression levels in flat or slightly flat surfaces; in the case of a complex 3D model, instead, the choice of a format became important. Across all tests, processing times were found to also play a key role, especially in point cloud generation. The qualitative and quantitative analysis, carried out on the different orthophotos, allowed to highlight a modest impact in the use of the TIFF format and a strong influence as the JPEG compression level increases.

## 1. Introduction

UAV photogrammetry describes photogrammetric measurement platforms that operate either remotely controlled, semi-autonomously or autonomously, all without a pilot, as well as photogrammetric processing of images obtained by UAV (Unmanned Aerial Vehicle) platform [1]. Recently, the combination of images obtained by UAV platforms and algorithms based on the structure from motion (SfM) and multi-view stereo (MVS) algorithms has become widely popular because it is possible to build 3D model or orthophoto in rapid, automatic and easy way. The fields of application of photogrammetry using UAV platforms are numerous: coastal mapping [2], archaeological environment [3,4], 3D reconstruction of simple and complex architecture [5], in civil engineering applications [6] or in disaster management and civil security applications [7] and in natural environment monitoring [8]. Sensors mounted on UAV platforms for photogrammetric purpose may be of different types (dSLR—digital single-lens reflex, compact or action camera, etc.).

In general, the images acquired with these passive sensors may be stored as compressed or uncompressed. Data compression refers to the process of reducing the amount of data required to represent a given quantity of information [9]. Therefore, image compression ratio can be defined as the ratio of the number of bytes of the original image before compression to the number of bytes of the compressed image [10]. Image compression tasks can be accomplished by the use of coding methods, spatial domain compression, transform domain compression or a combination of these methods [11,12]. In image processing fields, there are two main ways to compress images: lossless and lossy compression (or irreversible compression) [13]. In lossless compression schemes, the reconstructed image is exact replica of the original image. In lossy image compression, the reconstructed image contains degradation relative to the original. In lossy compression, higher compression can be achieved when compared to a lossless compression method.

Since a lot of photos are needed to build a three-dimensional model by photogrammetry and multi-view stereo approach, in general the users tend to compress the images in order to increase ease of management. Consequently, the impact of the compression of the images was evaluated over the years. Maeder [14] wrote at compression rates of less than 10:1, matching accuracies which generate a range of 5% to 10% discrepancies over the whole image can be achieved on substantially different image data. Zhilin et al. [15] wrote about the effects of JPEG compression on the accuracy of Photogrammetric Point Determination (PPD) by empirical investigation. In this case study, in processing various JPEG compressed images, the accuracy of the more coordinates of the pass points was assessed and compared with that obtained from the original images (i.e., without compression). The empirical results show that, when the compression ratios are under the threshold value of 10, the compressed image is near-lossless, i.e., the visual quality of JPEG compressed images is still excellent, and the accuracy of manual image measurement is essentially not influenced. Shih and Liu [16] evaluate the effects of JPEG 2000 compression on automated digital surface model (DSM) extraction, using the area-based matching technique. The authors, using two stereo pairs of aerial photographs (film camera) show that the DSM obtained from image quality based on the JPEG 2000 compression are clearly superior to the commonly used JPEG compression technique, especially for high compression ratios. Akca and Gruen [17] examine the potential of mobile phones to be used as front-end sensors for photogrammetric procedures and applications and using JPEG compression images. In this study, the transformation from TIFF to the JPEG format involved in a slight loss of accuracy both from a planimetric and altimetric point of view. O’Connor [18] discusses the impact of image quality on SfM Photogrammetry varying the color, compression and noise. In particular, in the Hunstanton study, the compression images task produced a median error of 6.49 mm as opposed to the 6.22 mm than RAW images. Pepe et al. [19], using airborne images generated by nadir-oblique cameras and SfM-MWS approach, wrote that the compression of the image produces an error on the point cloud variable according to the compression ratio. Indeed, by the comparison between the point clouds generated using TIFF image (reference) and the several point clouds generated using a different value of JPEG compression value, the authors were able to estimate a difference that increases respect to the compression level and reaches values of about 0.6 m. Kiefner and Hahn [20] wrote about the impact of different compression algorithms on image matching quality theoretically and experimentally; in particular, representative algorithms of three different compression methods, the JPEG, Wavelet and Fractal compression, are used to verify and quantify the theoretically found relation between matching accuracy and compression ratio. In this latter study, it is shown that Wavelet compression influences the compressed images less that JPEG and Fractal compression. Marčiš and Fraštia [21] empirically analyze the influence of compression of various images on the measurement accuracy of points in the image plane and the resulting 3D coordinates in the reference system. Re et al. [22] analyze the compression effect with respect to two main aspects that have to be taken in account during the analysis and the DTM generation: the texture of the image (local contrast and image content) and the surface characteristics of the acquired features.

In this study, the influence of the several format of images and level of JPEG compression in UAV photogrammetry and, of consequence, in applications where object-camera distance is quite close was evaluated focusing upon three main aspects: (i) accuracy in alignment of the SfM approach; (ii) the difference between the several 3D point cloud and (iii) quality of the orthophoto. To achieve this aim, three case studies were taken into consideration in order to analyze the different morphology of the area to be investigated: one test site is characterized by a flat ground; another slightly steep ground; a third area concerns the remains of a *hexastyle peripteral* Greek temple of the sixth century BC.

## 2. Characteristics of Some Digital Image Formats Used in Photogrammetry

### 2.1. RAW

A camera raw image file contains minimally processed data from the image sensor of either a digital camera. Indeed, the name “RAW” indicates that the image captured by the CCD (charge-coupled device) or CMOS (complementary metal-oxide semiconductor) sensor of the camera is recorded in its original and numerical form, i.e., after it has only been converted from analogic to digital, without further processing by the camera. Due to the fact that they offer greater creative control, raw file formats are popular in digital photography. However, cameras can use many different raw formats, the specifications for which are not publicly available. This means that not every raw file can be read by a variety of software applications. As a result, the use of these proprietary raw files as a long-term archival solution carries risk and sharing these files across complex workflows is challenging. For this reason, a new file format was developed and called digital negative (DNG) by Adobe Company, San Jose, CA, USA. This addresses the lack of an open standard for raw files created by individual camera models and ensures that users easily access their files. Hence, DNG is a publicly available archival format for raw files which are generated by various digital cameras. An advantage found in the DNG format compared to the proprietary raw format, is that in addition to the compatibility advantage, the use of DNG also saves a little space: an image in DNG format is on average 10% to 20% smaller than the proprietary raw format.

### 2.2. TIFF

The Tagged Image File Format, also called TIFF, is one of the most popular and flexible of the current public domain raster file format developed by Aldus Corporation [23]. To date, TIFF is a registered trademark of Aldus, but belonging to the Adobe company. The word “Tagged” in “TIFF” refers to this format’s complicated file structure. The initial header of the file data is followed by “chunks” of data called “tags,” which convey the image information to the program displaying the file. Indeed, the actual TIFF specifications define over 70 different tag types [24]. This means that the level of complexity allows great flexibility between viewers.

TIFF uses lossless compression to maintain image integrity and clarity and are often used for photogrammetric purposes or professional photography. For TIFF images, different compression formats are possible: LZW (Lempel–Ziv–Welch) and ZIP are lossless, that is without information loss while Lossy JPEG, i.e., with information loss. In the present study, we used a TIFF format without any compression and without resizing the image compared to the original format.

### 2.3. JPEG

JPEG Compression is the name given to an algorithm developed by the Joint Photographic Experts Group whose purpose is to minimize the file size of photographic image files. The JPEG compression principle is the use of controllable losses to reach high compression rates. In this context, the information is transformed to the frequency domain through DCT (discrete cosine transform) [25]. Since the neighboring pixels in an image have a high probability of showing small color variations, the DCT output will group the highest amplitudes in the lower spatial frequencies. The JPEG compression can be divided into five main steps: color space conversion, down-sampling, 2-D DCT, Quantization and entropy coding. The first two operations are used only for color images while for gray scale image we use only the last three steps.

Photoshop software provides compression levels from 0 to 12. A setting of 12 will apply the least amount of compression and give the highest image quality; a setting of 0 will apply the greatest amount of compression and be the most lossy [26]. These values are related to the compression rate, as shown in the following table (Table 1) [27].

## 3. Data and Methods

### 3.1. Method

In general, the images acquired by the UAS (Unmanned Aircraft Systems) are stored in DNG format. Since it is possible to transform the DNG image in TIFF and JPEG format using raster graphics editor software, such as Adobe Photoshop CC 2015 (developed and published by Adobe, Inc.), more datasets were built:

1. Dataset containing DNG images;
2. Dataset containing TIFF images;
3. Dataset containing JPEG images with compression equal to 1, i.e., high level of compression (called JPEG1 in the study);
4. Dataset containing JPEG images with compression equal to 6, i.e., medium level of compression (called JPEG6 in the study);
5. Dataset containing JPEG images with compression equal to 12, i.e., low level of compression (called JPEG12 in the study).

In Photoshop environment, the “Image Processor” script allows the image format conversion process in automatic way: in fact, it is necessary to simply specify the input and output folder and the desired file format. In the specific case, the original DNG format was converted to JPEG format indicating the three different compression levels taken into consideration (1, 6 and 12).

As far as the conversion to the TIFF format is concerned, the process took place in the same way described above, excluding the LZW compression option (the algorithm that does not lead to the loss of any part of the original information during the compression phase of the data itself).

The ICC (International Color Consortium) profile, i.e., the numerical representation of colors in a given “color space”, remained incorporated in both compression cases, thus allowing colors to be preserved when switching from one format to the other.

The image orientation based on the SfM approach has become quite popular in close-range photogrammetry since that it may solve the camera’s orientation in a fully automatic way, without any a priori knowledge of the approximate positions for cameras and 3D points. Therefore, considering a series of 2D images acquired from different observation points, by the use of SfM approach, it is possible to obtain the reconstruction of three-dimensional models and the production of sparse point cloud [28]. After this step, it is possible to run a dense reconstruction phase using Multi-View Stereo (MVS) [29]; this latter step allows obtaining a dense point cloud. In general, the several processing steps that lead to the construction of the model and implemented in many commercial software are: (i) alignment of the images; (ii) building a dense point cloud (PC) and (iii) building mesh [30]. In addition, if a (2D) orthophoto is required, once having chosen the suitable plane of projection, it is possible to build the orthomosaic. The post-processing of the dataset is performed in Agisoft Metashape environment; the setting “highest” can be chosen in order to work with a full resolution photo. In addition, to evaluate the independence of the algorithms implemented in the software, a further analysis was conducted using the 3DF Zephir aerial software. The elaborations in the latter software must be performed by setting the equivalent processing parameters present in Agisoft Metashape.

The first step of the photogrammetric processing concerns the construction of the flight planning. The flight planning must be designed in such a way that the images have a high overlap between them; for example, typical characteristics values for nadir photos are: 60% of sidelap and 80% of endlap [31]. If the block geometry is adequate, camera calibration parameters may also be estimated within the bundle adjustment that is integrated into the SfM pipeline, whose core is the automatic extraction and robust matching of corresponding features from a set of multiple overlapping images [32].

The several datasets may be processed according to the photogrammetric pipeline and, at the end of the process, to perform an evaluation about the accuracy of the single dataset. In addition, it is possible to make a comparison between the results obtained with the DNG dataset and those with a different format and image compression level.

The evaluation of the accuracy on 3D model reconstruction is evaluated by the following aspects:

1. quality of the alignment of the images (error pixel);
2. estimation of errors through comparison with points use ground control points (GCPs) obtained by topographical way;
3. comparison between the point cloud generated by DNG image format and the other point cloud generated using TIFF and JPEG formats;

The additional parameter taken into account affecting the reconstruction of 3D models concerns the time processing. In this way, a quantitative comparison in the several step of the reconstruction of 3D models between the several dataset and test sites is performed.

As regards the evaluation on the quality of the orthophoto may be performed by a qualitative and quantitative approach. The first approach consists of examining the image quality from a visual point of view by examining certain subjective parameters, such as brightness, exposure, contrast, etc. The quantitative approach, on the other hand, takes into account that is based on the use of some indexes. In this study, three indices were considered: RMSE (root mean square error), RASE (relative average spectral error) and ERGAS (erreur relative global adimensionnelle de synthèse). Root mean square error (RMSE) index is computed using the formula [33]:

(1) $$RMSE\left(band_{k}\right)=\sqrt{BIAS_{k}{}^{2}+\sigma_{k}{}^{2}}$$

where BIAS is the difference between the mean values of the reference orthophoto (DNG) and the other orthophoto; $\sigma_{k}$ standard deviation of the difference orthophoto (DNG) and the other orthophoto.

Relative average spectral error (RASE) index characterizes the average performance of a method in the considered spectral bands which is calculated including all the single channel (red, green and blue) by following formula [34]:

(2) $$RASE=\frac{100}{M}\sqrt{\frac{1}{n}\overset{n}{\underset{i=1}{\sum }}RMSE_{i}^{2}}$$

where M is the mean value of Digital Numbers (DNs) of the n input orthophotos (images).

The ERGAS (erreur relative global adimensionnelle de synthèse), also indicated as a dimensionless global relative error in synthesis and introduced by Wald [35], is another index to evaluate the comparison between two images using the following formula:

(3) $$ERGAS=100\frac{h}{l}\sqrt{\frac{1}{N_{Bands}}\overset{N_{Bands}}{\underset{k=1}{\sum }}{\left(\frac{RMSE\left(Band_{K}\right)}{MS_{k}}\right)}^{2}}$$

where h spatial resolution of reference (orthophoto) image; l spatial resolution of the orthophoto obtained with other format or JPEG compression; $N_{Bands}$ number of bands of the reference image; $MS_{k}$ mean radiance value of the k-th band of MS image.

This latter index is widespread in data fusion applications, such as the pan-sharpening [36], since that allows comparing images at different spatial resolutions, as for example in the case of satellite images where the spatial resolution of the panchromatic band has a higher resolution than multispectral. Low values of RMSE, RASE and ERGAS index indicate good quality between the images compared; in the ideal transformation, these indexes should be close to zero.

**Figure 1:**
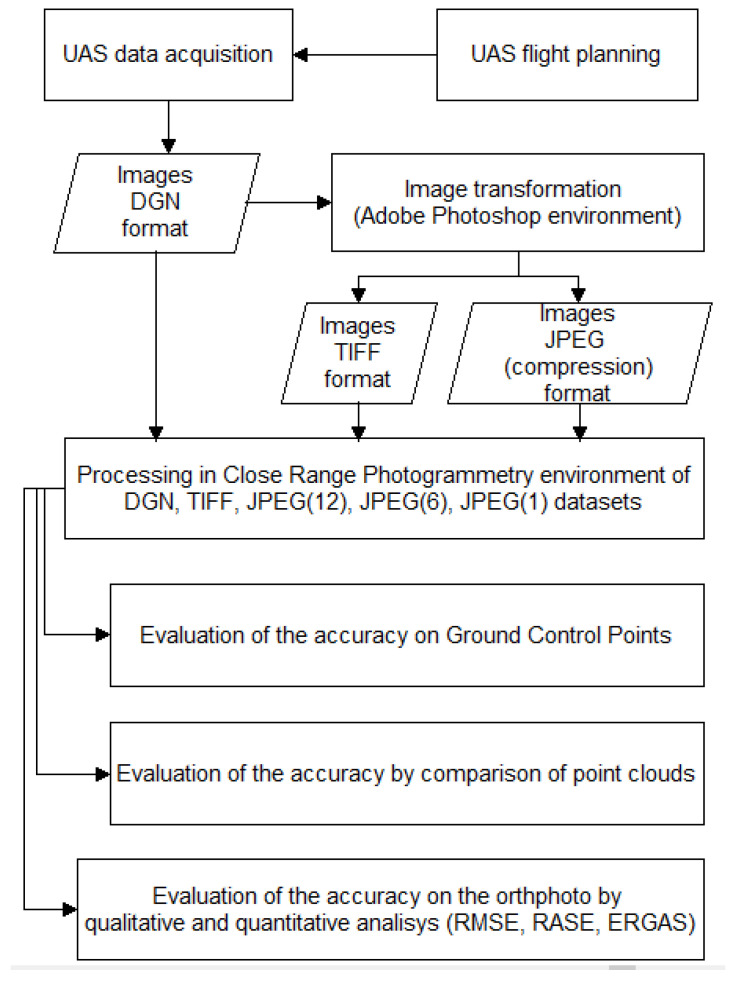
Pipeline of the developed method of investigation.

Therefore, the several aspects of the research that we want to investigate can be summarizing in the following pipeline (Figure 1).

### 3.2. UAV and Camera Features

In all case studies, DJI Mavic 2 Pro, developed by DJI Company, Shenzhen, China, was used. DJI Mavic 2 Pro, a popular consumer UAV (Quadcopter) fitted with a high-resolution color camera. Indeed, DJI Mavic 2 Pro, featuring the collaboratively developed Hasselblad L1D-20c, brings innovative experiences to the field with advancements in drone photography and UAV photogrammetry. The Hasselblad L1D-20c allows the user to obtain a higher standard for aerial image quality. A fully stabilized 3-axis gimbal with its powerful 20MP 1” sensor, it offers improved lowlight shooting capabilities in comparison to other drone cameras. The main features of this system are reported in Table 2.

The remote controller works at both 2.4 GHz and 5.8 GHz; due the special transmission technology developed by DJI Company, it is possible to transmit data up to distance of 8 km and is able to display video from the UAV on the mobile device with a resolution up to 1080p.

## 4. Empirical Tests

### 4.1. Mission Planning

**Figure 2:**
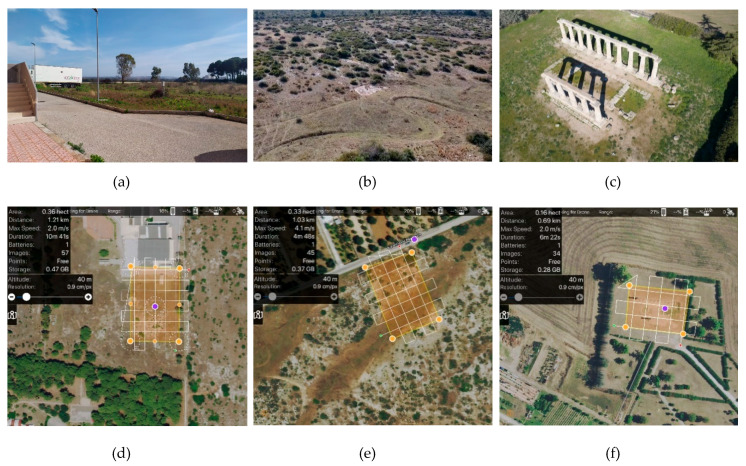
Test sites under investigation: panorama image of the test site 1 (**a**), test site 2 (**b**) and test site 3 (**c**) flight planning on test site 1 (**d**), on test site 2 (**e**) and on test site 3 (**f**).

The experimentations were carried out on three test sites:

1. Test site 1 (Figure 2a), a flat green area near the structure of the Polytechnic of Bari in Taranto headquarters (Italy);
2. Test site 2 (Figure 2b), a natural river bed with important slopes;
3. Test site 3 (Figure 2c), a Cultural Heritage site located in Metaponto (Italy), the so called “Tavole Palatine” are the remains of a Greek temple of the sixth century BC, dedicated to the goddess Hera.

The flight planning was designed in Map Pilot for DJI app, which enabled to build and to obtain optimal flight path by the use of the Maps Made Easy processing service. In particular, the flight planning, using a grid path, were designed in order to obtain the following parameters: flight altitude of 40 m AGL (Average Ground Level) or better of a GSD equal to 0.009 m, an overlap value of 80% and a sidelap value of 60%. The flight planning on the three test sites, with the characteristics of the area under investigation, are reported in Figure 2d–f. Furthermore, in the test site 3, 45° oblique images with a 60% overlap were added in order to ensure complete coverage of the shaft, capital and architrave of the temple.

The first two photogrammetric flights (test site 1 and 2) were carried out in the absence of wind and favorable light conditions; in test site 3, on the other hand, the flight was carried out at such a time as to have lighting conditions that could generate natural shadows due to the elevated elements that formed the ancient temple.

### 4.2. Post-Processing of the Datasets and Evaluation of the Accuracy on GCPs

In order to evaluate the accuracy of the photogrammetric process, simple panels in black-white were used as GCPs. These points may be determined by traditional topographic survey or GNSS (Global Navigation Satellite Systems) techniques. In all datasets, a GNSS survey (in static way) with dual frequency Leica GS12 was carried out. In addition, to estimate with high accuracy the spatial coordinates of the GCPs, GNSS data from at least two permanent stations (master) belonging to HxGN SmartNet CORS (Continuously Operating Reference Station) were used. In the three case studies, the maximum distance achieved between master-rover was 18 km. The post-processing of the GNSS data, i.e., the differential GNSS, was carried out using Leica GeoOffice (LGO) v. 8.2. In this way, the coordinates of the GCPs were determined with an accuracy lower 1 cm.

#### 4.2.1. Post-Processing in Metashape Software

The datasets of the images were elaborated by the use of Metashape Agisoft. The total error (TE), i.e., the Standard Deviation (SD) value for X, Y, Z coordinates for all the cameras, was evaluated on GCPs and CPs (Check Points) by the following formula:

(4) $$TE=\sqrt{\overset{n}{\underset{i=1}{\sum }}\frac{{\left(x_{i,est}-x_{i}\right)}^{2}+{\left(y_{i,est}-y_{i}\right)}^{2}+{\left(z_{i,est}-z_{i}\right)}^{2}}{n}}$$

The results of the processing, evaluated on GCPs, are reported in Table 3, Table 4 and Table 5. In addition, in these latter tables, we have reported the number of Tie points obtained in the several datasets. In Table 6, Table 7 and Table 8, instead, we have reported the SD values evaluated on the CPs.

Once this process was completed, it was possible to build the dense point cloud of each dataset. In particular, the setting “low” quality (images are downscaled before the dense matching procedure by a factor of 12.5%) was used in order to generate the dense point cloud. The number of the point cloud generated in the several formats and on the several test sites are demonstrated in the following Table 9.

#### 4.2.2. Post-Processing in 3DF Zephir Software of the Datasets Containing Images Acquired on the Test Site 3

Since the site 3 test is more complex from a computational point of view, the dataset was processed 3DF Zephir software in different formats and different levels of JPEG compression. Table 10 and Table 11 show the results of the elaborations in 3DF Zephir, where it is possible to note as, the RMSE values, are comparable with those obtained with Agisoft Metashape. As far as the number of tie points and dense point clouds generated by 3DF Zephir is concerned, they are lower than those generated by the other software considered.

### 4.3. Orthophoto Generation

**Figure 3:**
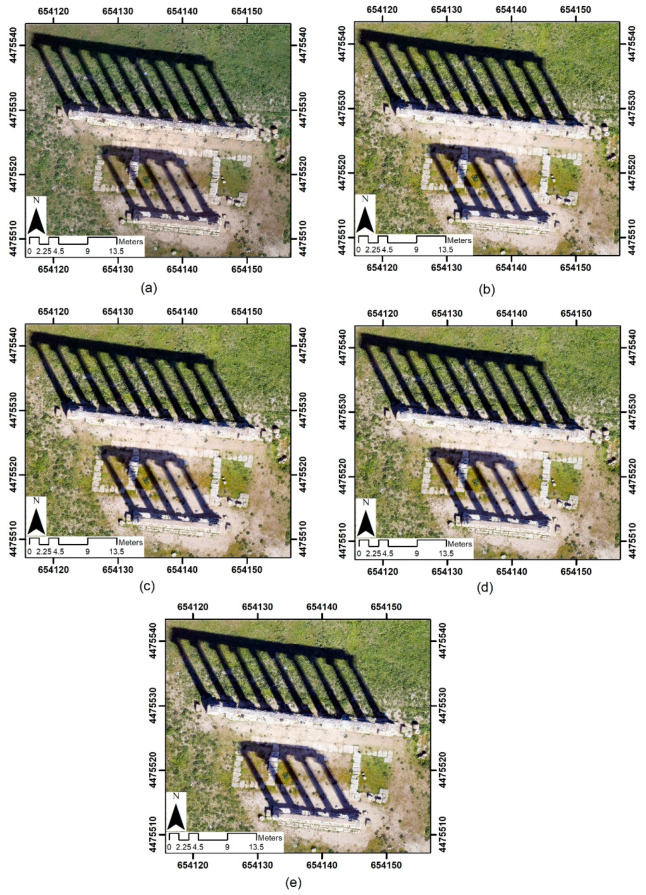
Orthophoto of the test site 3 using different image formats: digital negative (DNG) (**a**); TIFF (**b**); JPEG at low level of compression (JPEG12) (**c**) JPEG at medium level of compression (JPEG6) (**d**) and JPEG at maximum level of compression (JPEG1) (**e**).

In general, after you have built the mesh, it is possible to obtain the orthophoto (of course you need a suitable plane, such as the horizontal one). Since the images obtained in test site 3 also contain shadows coming from the structures in elevation, the decision was made to generate the orthophotos only of the case study concerning the old temple and, consequently, to evaluate the impact of the choice of the type of format and the level of JPEG compression of the images in the construction of the orthophoto. An orthophoto with a GSD of 0.03 m for each image dataset was built, as shown in Figure 3. It is evident from observation of the orthophotos (see Figure 3) that the image obtained from the JPEG1 dataset is of lower quality than the others.

## 5. Evaluation of the Accuracy

### 5.1. Comparison between the Point Clouds

**Figure 4:**
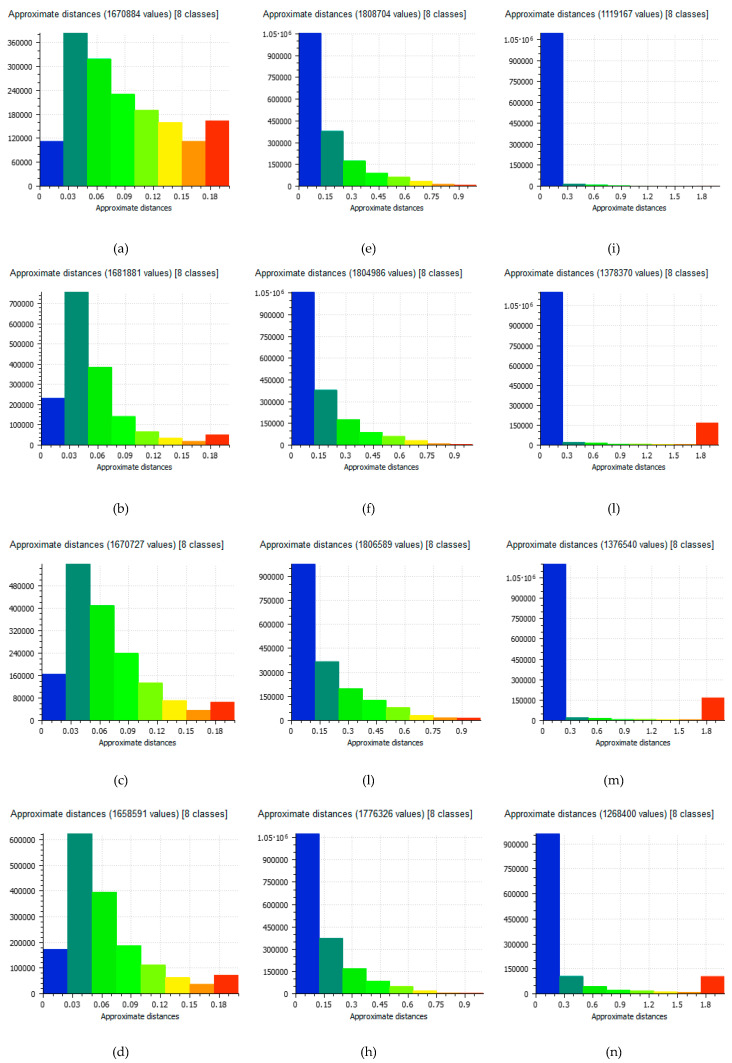
Histograms of difference between the point cloud generated by DNG images and the other point clouds generated by several format e level of JPEG compression: test site 1: TIFF (**a**), JPEG12 (**b**), JPEG6 (**c**), JPEG1 (**d**); test site 2: TIFF (**e**), JPEG12 (**f**), JPEG6 (**g**), JPEG1 (**h**); test site 3: TIFF (**i**), JPEG12 (**l**), JPEG6 (**m**), JPEG1 (**n**).

The point cloud generated by the use of DNG file format represents the reference dataset and, of consequence, it was possible to perform a comparison between the several datasets. Cloud Compare software was used for the several tests. The results of the comparisons, using histograms, is shown in Figure 4. In particular, the first column shows the values obtained in the test site 1, the second column shows the values obtained in the test site 2 and the third column shows the values obtained in the test site 3.

From Figure 4, it is possible to note how in all case studies the difference between point clouds is contained in a few centimeters; the most substantial portion of the points illustrate a difference of about 1 cm on the test site 1 and 2. In the test site 3 (see Figure 4n), instead, the difference has become quite important compared to previous cases.

Subsequently, taking into account the statistical data obtained from each test, it was possible to calculate the mean and Standard Deviation value (Table 12).

It is possible to observe in Table 12 that as far as the mean value obtained from the comparison between the point clouds at different compression levels, compared to the one obtained with the DNG format, the JPEG 1 format (maximum compression), results to be the one with the maximum error value. Moreover, it is possible to notice that the dataset that gave the best response in the comparison between the point clouds was the JPEG 12 that kept, in the three different case studies, an average value comparable in any case in the order of about 1 cm. The data observed are confirmed in the standard deviation (see Table 12) where it is easy to see how the maximum dispersion value is attributable to the JPEG1 format, while the TIFF and JPEG12 formats are those that gave appreciable results especially in the area 3 test.

**Figure 5:**
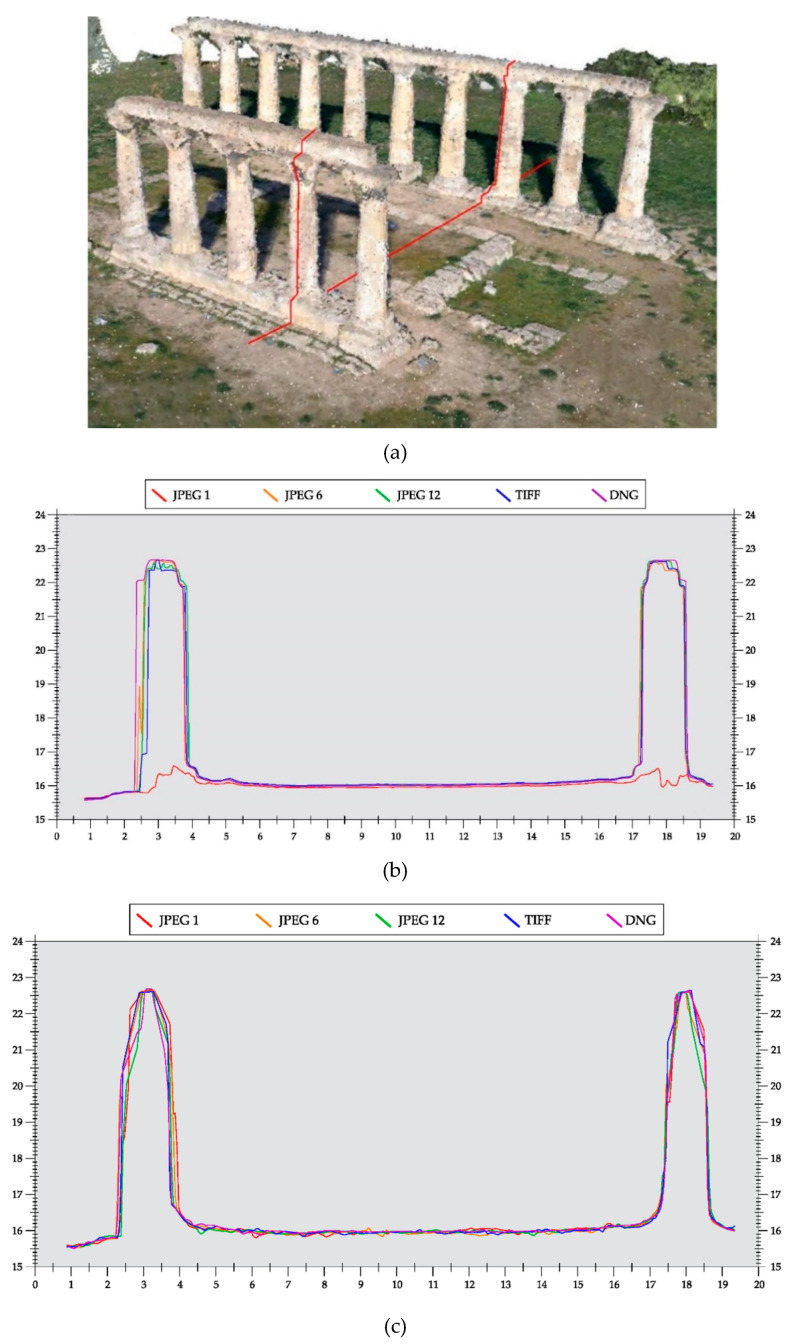
Comparison of point clouds in a section of the temple: (**a**) 3D point cloud with the identification of the section, (**b**) sections indicated with different color obtained from Agisoft Metashape, and (**c**) 3DF Zephir.

A further analysis of the point distribution generated with images in different formats and compression was conducted by extrapolating a section from the 3D point cloud model. A cross-section was considered (Figure 5a) of the case study concerning the remains of the temple.

By overlapping the profiles, it is possible to notice that, the more accurate profile and which faithfully reflects the original profile of the column of the temple, is the DNG; conversely, the JPEG at maximum compression (JPEG1) provides, in terms of accuracy, the worst result; even the column profile was not really generated in the dense point cloud. The average distance measured between the different profiles of the column is of the order of a few centimeters; on the columns-beams of the times, it also reached a maximum distance of 15 cm, while along the ground between the two columns maximum distance was about 5 cm. This means that in survey of 2.5D objects, the choice of an image format becomes important (Figure 5b).

**Figure 6:**
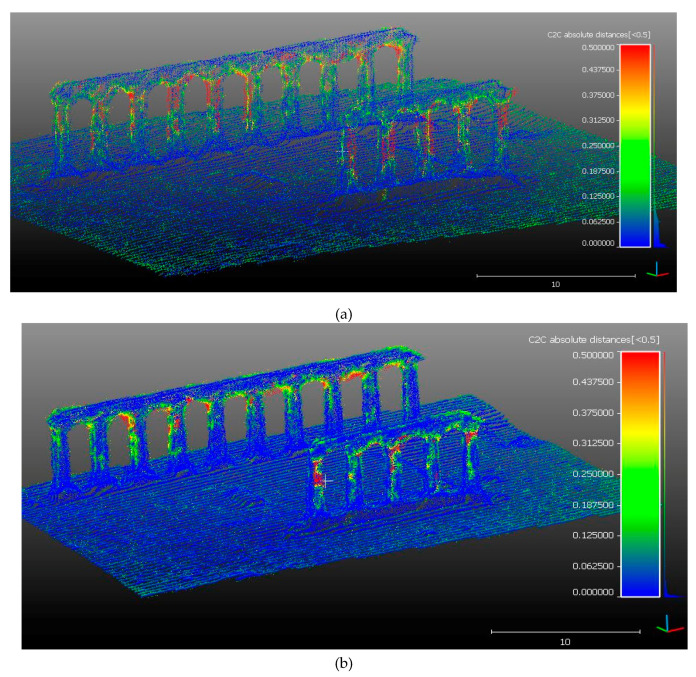
Comparison of point clouds (JPEG12) elaborated by Agisoft Metashape and 3DF Zephir: (**a**) low quality and (**b**) medium quality.

A further analysis was conducted on point clouds generated by the 3DF Zephir software. In the same way as the sections shown in Figure 6b, profiles of the point clouds generated with 3DF Zephir are shown in Figure 5c.

In this case, it was possible to obtain a profile of the columns also with the point cloud coming from images with low compression (JPEG1). The difference of the point clouds measured between the column profiles remains, however, in the order of a few centimeters confirming the importance of the image format in the object detection process (see Figure 5c).

In order to evaluate the accuracy in terms of dense point clouds, an analysis focused on the comparison between the dense clouds from Agisoft Metashape and 3DF Zephir Aerial, respectively. In particular, the point clouds compared were obtained from image processing in JPEG12 format. It can be seen that the two dense point clouds have a distance of no more than 6 cm in the reconstruction of the temple base and the surrounding terrain. Greater differences can be found, as shown in Figure 6a, in the definition of the profile of the column and some sections of the beams above. This means that 3DF Zephir software was able to describe in greater detail, through a dense point clouds, the geometry of the columns and their bases. In order to check if and how a higher point density can affect the distance between point clouds generated with two different software, the JPEG12 dataset were reprocessed using a “medium” quality according to the setting implemented within Agisoft Metashape. Of course, an equivalent setting was used in 3DF Zephyr software. The comparison of the dense point clouds shows a similar behavior between low and medium setting (Figure 6b).

### 5.2. Time Processing

A further aspect of the research concerned processing time in two fundamental stages: image alignment and dense cloud generation. Of course, the processing of the dataset was obtained using the same Personal Computer. The time needed for processing in the different case studies are shown in the following tables (Table 13, Table 14 and Table 15). In addition, we have calculated the normalized time than registration and dense point cloud generation obtained using DNG format of the images.

From the analysis of Table 13, Table 14 and Table 15, it is possible to see how the chosen image format and level of JPEG compression allows a time reduction in mean of the 13% in the alignment step and about the 33% in the generation of the dense point cloud. Therefore, processing times in point cloud generation play an important role.

### 5.3. Evaluation of the Quality of the Orthophoto

**Figure 7:**
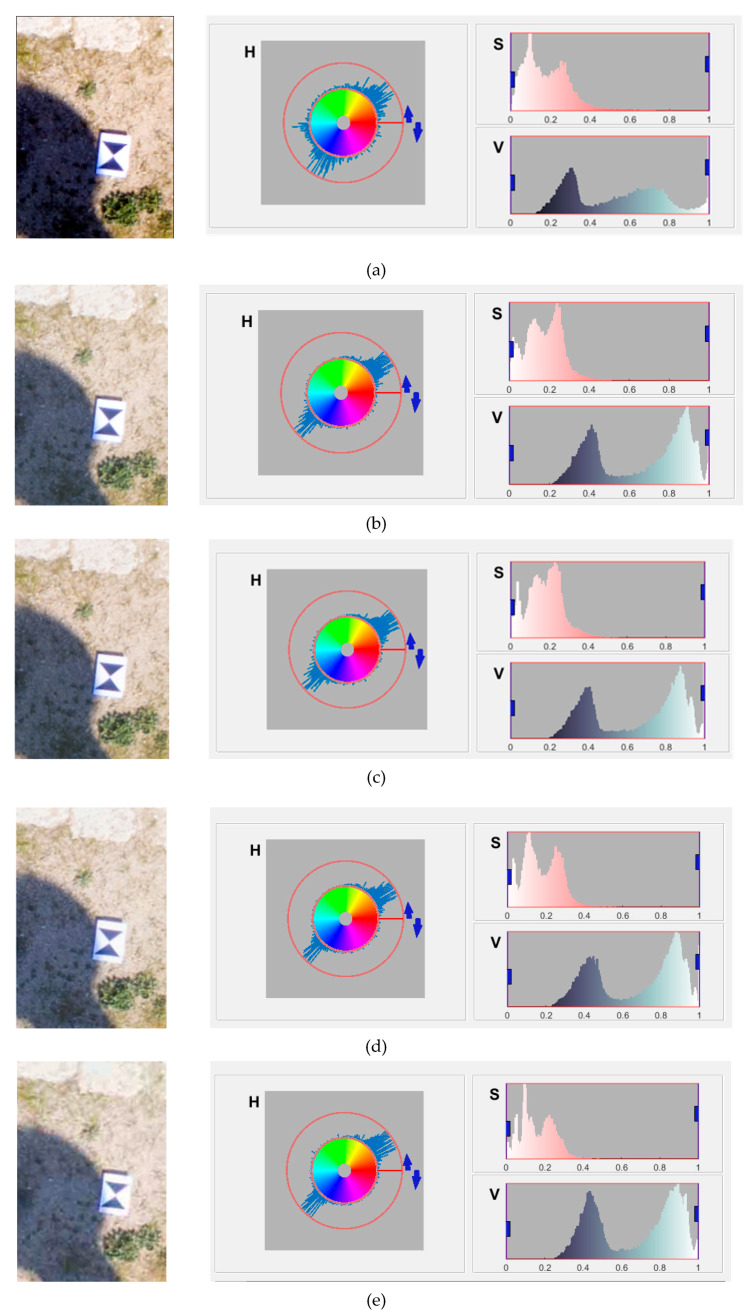
Particular of the several orthophoto and their color features: (**a**) DNG, (**b**) TIFF, (**c**) JPEG12 (**d**) JPEG6, (**e**) JPEG1.

In order to analyze the quality of the generated orthophotos used different formats and JPEG compression levels. A portion of the orthophoto was considered in a particular area that had characteristic elements (shadow, target, structures and small vegetation). Then, it was possible to obtain in Matlab software, a representation of each image in the color space of HSV (Hue, Saturation, Value components). HSV is a model particularly useful because it describes the colors in a way closer to the human perception. Indeed, Hue represents the color perception (red, yellow, green, blue and purple). The Saturation represents the “purity” of the color and varies from 0% to 100%: highest value represents the full saturation (100%), while the minimum (0%) is equivalent to the grayscale. The Value is the quality by which it is possible to distinguish a light color from a dark one and its value varies from 0% to 100%. The representation in HSV space color of each image is reported in the following image (Figure 7).

At a visual level, the most notable difference is that related to the coloring of the image that is perfectly saturated and with well-defined contours in the case of the DNG.

As the compression level increases, it is possible to see a loss of resolution certainly confirmed also by a low value of sharpness: the contrast and the chromatic value between the shadows, the target and the elements belonging to the surrounding environment are poorly defined; the saturation values are very low to create a disturbance that does not allow a clear distinction of the vegetation in the areas out of shadow (see Figure 7e).

The markedly superior result, in terms of fidelity and image quality, is represented by the TIFF and the JPEG 12 format because from a visual comparison with the DNG format, they present only an increase in the brightness value.

From the observation of Figure 7, it is possible to note:

1. the hue in the DNG image has more chromatic values, that is, it has a greater distribution of colors than the other images;
2. the pixels in the histogram of the value are distributed in the tonal range in a uniform way and do not present peaks in correspondence of the extreme values and consequently, the image presents a more correct tonalization; in comparison, as the JPEG compression increases, it is possible to notice how the curve of the value moves to the right creating an apparent overexposure of the image.

The quantitative analysis was carried out taking into consideration three indexes (RMSE, RASE and ERGAS) and the parameters color of each image were compared than the orthophoto in DNG format. The elaboration of these indexes were performed in Matlab software. The results of this step is summarized in the following Table 16.

Table 16 confirms the results of the qualitative analysis; above all, it is highlighted that the JPEG12 image is very faithful to the image in DNG format. This issue can be seen from the low values achieved in both the RMSE index, RASE and ERGAS. Of course, as the compression level of JPEG increases, there is a loss of quality. As for the TIFF format image, it has very low values of the three indices and close to those obtained with a JPEG image with a compression value of 12.

### 5.4. Testing for Image Analysis by Modulation Transfer Function (MTF)

**Figure 8:**
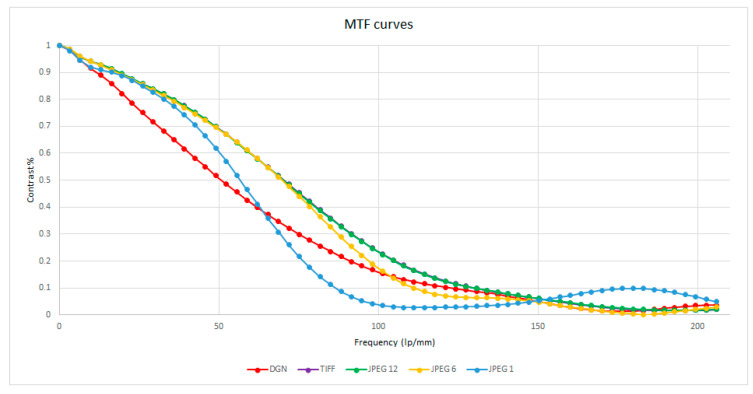
Modulation transfer function (MTF) curves.

To verify the sharpness on the images in relation to the compression levels and the different formats, we carried out a test using MTF Mapper software [37]. The MTF mapper package offers a collection of tools to measure modulation transfer function (MTF) values across edges in images. It computes the edge spread function of a step edge in an image, using a method similar to the one described by Khom [38]. Good indicators of image sharpness are spatial frequencies where MTF is 50% of its low frequency value (MTF50). The tests were conducted taking into consideration the black and white panel type ISO 12233 (standard for measuring the resolution of electronic imaging cameras). The ISO 12233 panel was photographed through the camera supplied with the drone mentioned above. The DNG file was transformed in the images with different formats and JPEG compression levels. Using the MTF mapper software, the MTF50 graph was generated taking into account the indicated point (in cyan color) of highest frequency. In this way, the different curves were extracted and superimposed in a graph (see Figure 8) where the contrast values are shown on the ordinates while the frequency values are shown on the abscissae, the latter expressed in lp/mm, i.e., line pairs for millimeter.

The curves shown in Figure 8 confirm the above, showing that as the compression level increases, there is a loss of sharpness especially at high frequencies. These results are consistent with what was stated in previous works [39].

## 6. Conclusions

The empirical results conducted on different test areas using UAV platform and with Hasselblad L1D-20c camera show that the choice of a type of TIFF and JPEG format with different compression values (value of the levels of compression values chosen: 1, 6 and 12) do not significantly affect the processing reconstruction of the 3D model compared to that obtained with images in DNG format; this issue was verified especially for rather flat surfaces. In fact, in the modeling of the temple columns (test site 3) the JPEG images with elevated compression (compression value equal to 1) produced point clouds that may not completely describe the geometry of the structure. The impact on geometry of the dense point clouds is more or less significant depending on the MVS-based approach algorithms implemented in the various commercial software.

The processing times required to obtain the 3D models are variable and related to the type of format and compression. In fact, especially in the generation of the dense cloud, the processing times vary significantly with the type of format and with the level of compression.

As far as the impact of the type of image on the generation of orthophotos is concerned, it was shown how the TIFF and JPEG with small value of compression (level 12) format allows the user to obtain results more faithful to the orthophoto generated with images in the DNG format.

Therefore, in light of the results obtained on the various empirical tests, both in terms of model accuracy and orthophoto quality, it was demonstrated that the best dataset to preserve the quality of the photogrammetric process (which uses images captured by UAV) is the one using JPEG images with a compression level of 12.

## Figures and Tables

**Table 1:** Compression levels available in Photoshop.

JPEG Compression	Photoshop Scale Name	Equivalent in%
0	Low	0–7
1	Low	8–15
2	Low	16–23
3	Low	24–30
4	Low	31–38
5	Medium	39–46
6	Medium	47–53
7	Medium	54–61
8	High	62–69
9	High	70–76
10	Maximum	77–84
11	Maximum	85–92
12	Maximum	93–100

**Table 2:** Features of the DJI Mavic 2 Pro.

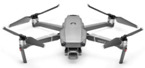	**Features**	**Specifications**
	*UAV Platform*	
	Max. take-off weight	907 g
	Maximum Speed (P-Mode)	48 km/h/13.4 m/s
	Flight time	~31 min
	*Camera: Hasselblad L1D-20c*>	
	Sensor	1″ CMOS; Effective pixels: 20 million
	Photo size	5472 × 3648
	Focal length	10.26 mm
	Field of view	approx. 77°
	Aperture	f/2.8–f/11
	Shooting speed	Electronic shutter: 8–1/8000 s

**Table 3:** Evaluation of the accuracy on GCPs and number of tie points—Test site 1.

Format	SD-GCPs					Tie Points
	TE (m)	X (m)	Y (m)	Z (m)	XY (m)	
DNG	0.054	0.006	0.006	0.053	0.009	93,311
TIFF	0.059	0.009	0.009	0.057	0.013	92,791
JPEG12	0.054	0.008	0.008	0.053	0.011	93,358
JPEG6	0.059	0.008	0.009	0.058	0.019	93,108
JPEG1	0.060	0.010	0.010	0.059	0.014	91,756

**Table 4:** Evaluation of the accuracy on GCPs and number of tie points—Test site 2.

Format	SD-GCPs					Tie Points
	TE (m)	X (m)	Y (m)	Z (m)	XY (m)	
DNG	0.045	0.025	0.031	0.020	0.040	66,092
TIFF	0.049	0.030	00.028	0.027	0.041	63,679
JPEG12	0.050	0.030	0.027	0.029	0.041	63,540
JPEG6	0.051	0.029	0.027	0.031	0.041	61,635
JPEG1	0.044	0.022	0.033	0.019	0.040	56,596

**Table 5:** Evaluation of the accuracy on GCPs and number of tie points—Test site 3.

Format	SD—GCPs					Tie Points
	TE (m)	X (m)	Y (m)	Z (m)	XY (m)	
DNG	0.013	0.008	0.007	0.008	0.011	167,627
TIFF	0.013	0.008	0.006	0.008	0.010	148,613
JPEG12	0.013	0.008	0.006	0.007	0.010	147,688
JPEG6	0.014	0.008	0.006	0.008	0.011	150,227
JPEG1	0.060	0.024	0.023	0.051	0.034	148,611

**Table 6:** Evaluation of the accuracy on control points (CPs)–Test site 1.

Format	SD-CPs				
	TE (m)	X (m)	Y (m)	Z (m)	XY (m)
DNG	0.055	0.006	0.006	0.054	0.009
TIFF	0.062	0.009	0.009	0.061	0.012
JPEG12	0.058	0.008	0.008	0.057	0.011
JPEG6	0.064	0.009	0.008	0.062	0.012
JPEG1	0.060	0.010	0.010	0.063	0.018

**Table 7:** Evaluation of the accuracy on CPs—Test site 2.

Format	SD-CPs				
	TE (m)	X (m)	Y (m)	Z (m)	XY (m)
DNG	0.049	0.027	0.034	0.022	0.044
TIFF	0.054	0.033	0.030	0.030	0.045
JPEG12	0.055	0.033	0.030	0.032	0.045
JPEG6	0.056	0.033	0.030	0.033	0.045
JPEG1	0.046	0.024	0.036	0.016	0.044

**Table 8:** Evaluation of the accuracy on CPs—Test site 3.

Format	SD-CPs				
	TE (m)	X (m)	Y (m)	Z (m)	XY (m)
DNG	0.016	0.010	0.007	0.010	0.012
TIFF	0.014	0.010	0.006	0.008	0.012
JPEG12	0.014	0.010	0.006	0.008	0.012
JPEG6	0.015	0.010	0.006	0.008	0.012
JPEG1	0.070	0.028	0.027	0.062	0.039

**Table 9:** Points of dense cloud in the several test sites.

Format	Dense Point Cloud		
	Test Site 1	Test Site 2	Test Site 3
DNG	1,767,190	1,889,492	1,226,600
TIFF	1,670,902	1,808,733	1,119,258
JPEG12	1,681,901	1,805,020	1,378,370
JPEG6	1,670,747	1,806,635	1,376,540
JPEG1	1,658,614	1,776,380	1,268,410

**Table 10:** Evaluation of the accuracy using 3DF Zephir on GCPs and number of tie points—Test site 3.

Format	SD—GCPs					Tie Points	Dense Point Cloud
	TE (m)	X (m)	Y (m)	Z (m)	XY (m)		
DNG	0.018	0.010	0.008	0.012	0.013	22,254	624,766
DNG	0.018	0.010	0.008	0.012	0.013	22,254	624,766
TIFF	0.016	0.009	0.008	0.010	0.012	16,936	668,490
JPEG12	0.017	0.008	0.010	0.011	0.013	19,417	670,026
JPEG6	0.019	0.011	0.010	0.012	0.015	19,551	669,359
JPEG1	0.047	0.014	0.011	0.043	0.018	17,092	680,969

**Table 11:** Evaluation of the accuracy using 3DF Zephir on CPs and number of tie points—Test site 3.

Format	SD–CPs				
	TE (m)	X (m)	Y (m)	Z (m)	XY (m)
DNG	0.018	0.011	0.009	0.011	0.014
TIFF	0.017	0.011	0.007	0.011	0.013
JPEG12	0.019	0.012	0.010	0.010	0.016
JPEG6	0.027	0.015	0.016	0.016	0.022
JPEG1	0.037	0.014	0.011	0.033	0.018

**Table 12:** Table of mean and standard deviation values of the dense cloud difference than DNG format.

	Test n.1		Test n.2		Test n.3	
	MEAN (m)	SD (m)	MEAN (m)	SD (m)	MEAN (m)	SD (m)
DNG—TIFF	0.015	0.074	0.022	0.178	0.004	0.044
DNG—JPEG12	0.005	0.043	0.004	0.159	0.005	0.007
DNG—JPEG 6	0.015	0.075	0.009	0.227	0.004	0.063
DNG—JPEG1	0.006	0.050	0.046	0.167	0.069	0.137

**Table 13:** Time processing for the experimentation on test site 1.

	SfM Building Blocks			Dense Point Cloud Generation		
	Matching	Image Orientation	Normalized Time vs. DNG	Depth Map	Dense Cloud	Normalized Time vs. DNG
DNG	8 m 59 s	3 m 41 s	(760 s)	8 m 48 s	1 m 13 s	(601 s)
TIFF	7 m 46 s	3 m 23 s	12%	6 m 20 s	23 s	33%
JPEG12	7 m 47 s	3 m 37 s	10%	5 m 40 s	26 s	39%
JPEG6	7 m 39 s	3 m 19 s	13%	6 m 7 s	23 s	35%
JPEG1	7 m 33 s	3 m 09 s	16%	5 m 31 s	22 s	41%

**Table 14:** Time processing for the experimentation on test site 2.

	SfM Building Blocks			Dense Point Cloud Generation		
	Matching	Image Orientation	Normalized Time vs. DNG	Depth Map	Dense Cloud	Normalized Time vs. DNG
DNG	9 m 46 s	1 m 39 s	(685 s)	9 m 07 s	1 m 15 s	(622 s)
TIFF	8 m 10 s	1 m 32 s	15%	6 m 12 s	23 s	36%
JPEG12	7 m 32 s	1 m 35 s	20%	6 m 01 s	26 s	36%
JPEG6	7 m 26 s	1 m 32 s	21%	5 m 56 s	23 s	37%
JPEG1	7 m 19 s	1 m 17 s	25%	5 m 58 s	23 s	37%

**Table 15:** Time processing for the experimentation on test site 3.

	SfM Building Blocks			Dense Point Cloud Generation		
	Matching	Image Orientation	Normalized Time vs. DNG	Depth Map	Dense Cloud	Normalized Time vs. DNG
DNG	27 m 42 s	2 m 08 s	(1790 s)	54 m 38 s	4 m 52 s	(3570 s)
TIFF	25 m 29 s	3 m 30 s	3%	40 m 23 s	4 m 53 s	24%
JPEG12	25 m 21 s	2 m 29 s	7%	40 m 34 s	3 m 06 s	27%
JPEG6	25 m 08 s	2 m 21 s	8%	43 m 38 s	3 m 18 s	21%
JPEG1	24 m 49 s	4 m 10 s	3%	34 m 52 s	5 m 24 s	32%

**Table 16:** Evaluation of the accuracy by the use of root mean square error (RMSE), relative average spectral error (RASE) and erreur relative global adimensionnelle de synthèse (ERGAS) indexes.

Format	Channel	Mean	Bias	RMSE	RASE	ERGAS
TIFF	red	160.43	67.43	10.97	7.937932	1.288985
	green	156.00	59.87	9.54		
	blue	150.40	51.17	15.69		
JPEG12	red	158.63	67,35	9.17	7.508253	1.253453
	green	153.49	59.46	8.40		
	blue	146.88	51.52	15.53		
JPEG6	red	160.87	66.82	11.40	8.783363	1.34298
	green	157.14	59.32	10,73		
	blue	154.52	49.41	18.14		
JPEG1	red	161.75	65.21	12.38	9.731192	1.407486
	green	158.29	58.26	12.30		
	blue	156.48	47.78	20.30		
